# Finale: impact of the ORCHESTRA/ENCORE programmes on Southern Ocean heat and carbon understanding

**DOI:** 10.1098/rsta.2022.0070

**Published:** 2023-06-26

**Authors:** Andrew J. S. Meijers, Michael P. Meredith, Emily F. Shuckburgh, Elizabeth C. Kent, David R. Munday, Yvonne L. Firing, Brian King, Tim J. Smyth, Melanie J. Leng, A. J. George Nurser, Helene T. Hewitt, E. Povl Abrahamsen, Alexandra Weiss, Mingxi Yang, Thomas G. Bell, J. Alexander Brearley, Emma J. D. Boland, Daniel C. Jones, Simon A. Josey, Robyn P. Owen, Jeremy P. Grist, Adam T. Blaker, Stavroula Biri, Margaret J. Yelland, Ciara Pimm, Shenjie Zhou, James Harle, Richard C. Cornes

**Affiliations:** ^1^ British Antarctic Survey, High Cross, Madingley Road, CB3 0ET Cambridge, UK; ^2^ Department of Computer Science and Technology, University of Cambridge, William Gates Building 15 JJ Thomson Avenue, Cambridge CB3 0FD, UK; ^3^ National Oceanography Centre, European Way, Southampton SO14 3ZH, UK; ^4^ British Geological Survey, Keyworth, Nottingham NG12 5GG, UK; ^5^ Plymouth Marine Laboratory, Prospect Place, Plymouth PL1 3DH, UK; ^6^ Met Office Hadley Centre, FitzRoy Road, Exeter, UK; ^7^ National Oceanography Centre, Joseph Proudman Building, 6 Brownlow Street, Liverpool L3 5DA, UK; ^8^ Department of Earth, Ocean and Ecological Sciences, School of Environmental Sciences, University of Liverpool, Liverpool, UK

**Keywords:** ocean heat, ocean carbon, Southern Ocean, ocean circulation, ocean–atmosphere fluxes, climate

## Abstract

The 5-year Ocean Regulation of Climate by Heat and Carbon Sequestration and Transports (ORCHESTRA) programme and its 1-year extension ENCORE (ENCORE is the National Capability ORCHESTRA Extension) was an approximately 11-million-pound programme involving seven UK research centres that finished in March 2022. The project sought to radically improve our ability to measure, understand and predict the exchange, storage and export of heat and carbon by the Southern Ocean. It achieved this through a series of milestone observational campaigns in combination with model development and analysis. Twelve cruises in the Weddell Sea and South Atlantic were undertaken, along with mooring, glider and profiler deployments and aircraft missions, all contributing to measurements of internal ocean and air–sea heat and carbon fluxes. Numerous forward and adjoint numerical experiments were developed and supported by the analysis of coupled climate models. The programme has resulted in over 100 peer-reviewed publications to date as well as significant impacts on climate assessments and policy and science coordination groups. Here, we summarize the research highlights of the programme and assess the progress achieved by ORCHESTRA/ENCORE and the questions it raises for the future.

This article is part of a discussion meeting issue ‘Heat and carbon uptake in the Southern Ocean: the state of the art and future priorities’.

## 1. Introduction

Climate change is one of the most urgent issues facing humanity and life on Earth. A critical gap in our understanding of the climate system concerns the uptake, distribution and storage of heat and carbon by the oceans. Over 93% of the extra heat now present in the Earth System because of global warming is stored in the ocean [1,2]. The 2012 Intergovernmental Panel on Climate Change (IPCC) Assessment Report 5 (AR5) described strong increases in the energy stored in both the upper (approx. 170 × 10^21 ^J) and deep ocean (approx. 80 × 10^21 ^J) since the 1970s [3] and the global ocean was understood to be the largest reservoir of carbon in the climate system having absorbed approximately 30% of anthropogenic CO_2_ emissions since the start of the industrial period [4], moderating the rate of warming in the atmosphere. The Southern Ocean has been shown to be disproportionately important in this context, accounting for an estimated approximately 50% of the oceanic uptake of anthropogenic carbon, and greater than 75% of the heat uptake [5]. This central climatic role was known to be a consequence of the Southern Ocean's unique pattern of circulation, and particularly its meridional overturning circulation [6].

Notwithstanding this, AR5 also recognized numerous critical gaps in our understanding of the variability, evolution, underpinning processes and global impact of the Southern Ocean, both in terms of spatio-temporal observational gaps, and in terms of our ability to understand and model key processes. The need for large scale, coordinated examination of these missing pieces in our understanding was recognized, and in 2015 the UK's Natural Environment Research Council (NERC) commissioned the ORCHESTRA (Ocean Regulation of Climate by Heat and Carbon Sequestration and Transports) programme through National Capability. It was designed to integrate the expertise and capabilities of seven UK research centres, and to provide a flagship climate research programme centred around improving our understanding of heat and carbon in the Southern Ocean. Led by the British Antarctic Survey (BAS) it incorporated personnel, expertise and infrastructure from the National Oceanography Centre (NOC), Plymouth Marine Laboratory (PML), British Geological Survey (BGS), Centre for Polar Observation and Modelling (CPOM) and the Sea Marine Research Unit (SMRU) at the University of St. Andrews, and involved close collaboration with the United Kingdom Met Office. The project ran from 2016 to 2021, and was succeeded by the 1-year extension programme ENCORE (ENCORE is the National Capability ORCHESTRA Extension) from 2021 to 2022.

The overarching objective of ORCHESTRA/ENCORE (hereafter O/E) was to advance our understanding of the strength, variability and controls of the Southern Ocean uptake, storage and export of heat and carbon, and improve our capability to predict its climatic impacts. This was to be achieved through an ambitious combination of observations, modelling and novel analysis of existing datasets. O/E has since led to the collection of more than 1700 individual data series including: 12 hydrographic cruises, greater than 600 CTD casts, 186 mooring data series, 28 aircraft flight datasets, six autonomous ocean glider deployments, 1600 autonomous Argo profiles, 170 TB of data from newly developed high-resolution models, two gridded bathymetry products and a new suite of analysis tools for air–sea flux datasets. This landmark programme has driven a step change in our understanding of several aspects of Southern Ocean dynamics with over 100 peer-reviewed articles to date. It has made significant contributions to international assessments including the IPCC Special Report on the Ocean and Cryosphere in a Changing Climate (SROCC) and AR6, and supported and contributed to numerous other national and international research programmes, scientific coordinating bodies and policy makers. Its findings and commitment to open-access data will continue to shape the field for years to come.

This article reviews key findings and highlights of O/E, as well as updating previously published analyses and presenting selected new findings. The experimental design of O/E is described in the Methods section below, followed by a summary of selected highlight findings in the Results section. The wider relevance of O/E analysis and the new questions raised by this research are explored in the Discussion and Conclusion sections. The treatment of such a large programme in a single overview must necessarily be superficial, so wherever possible the reader is referred to more detailed O/E articles. This article serves to draw together the collective output of O/E and signpost the highlights of the programme, and to demonstrate the wider scientific and socio-political impacts that a coordinated programme of this scale can achieve.

## 2. Methods

O/E was broadly divided into three thematic areas of analysis: air–sea fluxes; surface layer to ocean interior processes; and basin-scale storage, transport and export. To address these questions, it explicitly incorporated observational components, high-resolution model development, the creation of new data products and analysis software, and coupled climate model analysis. Although each element of O/E had self-contained scientific goals, their objectives and methodologies were constructed to exploit complementarities and enable a holistic view of the Southern Ocean physical progresses controlling heat and carbon budgets to emerge. All observational datasets and selected model output are or are being made openly accessible via the British Oceanographic Data Centre and the Centre for Environmental Data Analysis.

### (a) Observational methodology

**Figure 1.**
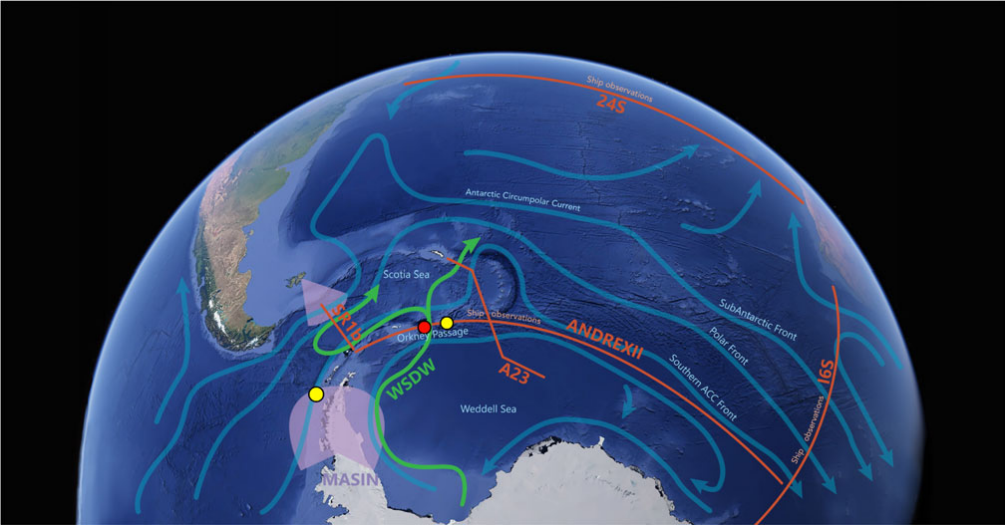
Schematic demonstrating primary regions of O/E fieldwork, overlaid with approximate pathways of the major regional ocean circulation features. Red lines indicate ship hydrographic sections (see electronic supplementary material, table S1), pink shading approximate areas of operation for MASIN flights. Yellow circles indicate the locations of the two major processes studies and red circle the location of the mooring array. WSDW indicates the approximate export pathway of Weddell Sea Deep Water through Orkney Passage. Graphic design by Ralph Percival. (Online version in colour.)

Observational elements focused on the UK operational region of the South Atlantic, notably the Weddell and Scotia seas (figure 1), which overlap with a significant component of the Southern Ocean meridional overturning circulation. O/E supported continuing repeat observations of high-frequency (A23, SR1b) and decadal (24°S, ANDREX) historical sections, expanding and extending an existing mooring array, process studies along ocean fronts and above sea mounts, aerial observations and the deployment of numerous float types.

#### (i) Hydrographic sections

**Figure 2.**
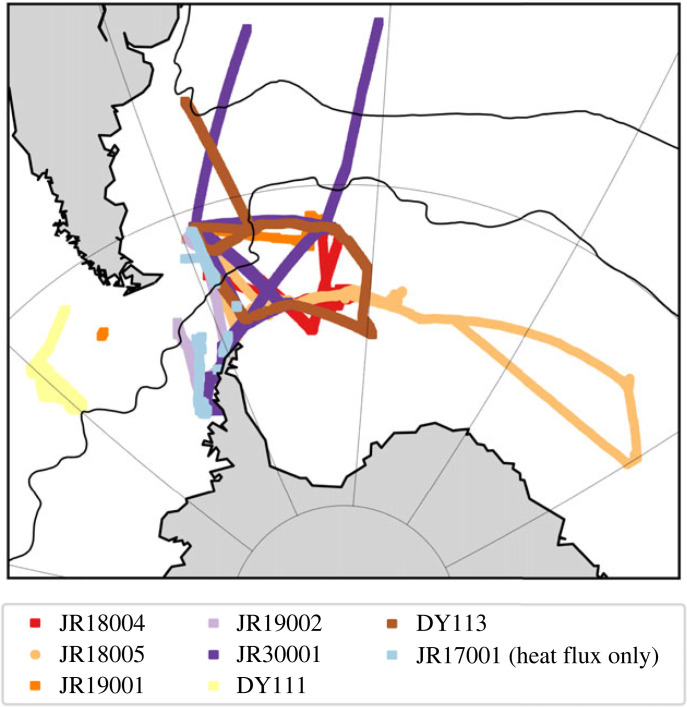
Location of air–sea heat and carbon flux measurements obtained using eddy covariance instrumentation during O/E. Ship tracks labelled by voyage number (see electronic supplementary material, table S1 for details). (Online version in colour.)

The ship hydrographic campaign incorporated 12 voyages of the RRS *James Clark Ross* (JCR), RRS *James Cook* and RRS *Discovery* (electronic supplementary material, table S1). Expeditions were designed such that, with the addition of the 2019 US-occupied I6S section at 30°E, sections with a full suite of hydrographic parameters (GO-SHIP recommended Level 1 parameters and more) occupied over a 1-year period would bound both the South Atlantic (south of 24°S) between Drake Passage and 30°E. This would permit the numerical inversion of the boundary fluxes to estimate property transports, storage, vertical transport and surface fluxes and comparison of O/E epoch budgets to those of a decade prior (e.g. [7–9]). The ANDREXII line along the northern boundary of the Weddell Gyre would allow the further decomposition of the domain into Weddell Sea and South Atlantic ‘boxes’. The annual SR1b and A23 sections maintained or enhanced the frequency of occupation of these key GO-SHIP hydrographic sections. All voyages collected water column oxygen (δ^18^O) isotopes, while several including all ‘box’ boundaries also included transient tracers (funded by UK programme TICTOC), carbon isotopes, and other enhanced sample collection (electronic supplementary material, table S1). Many of the voyages (figure 2) also carried the PML's autonomous eddy covariance system [10] mounted on the foremast of the ship at 21.5 m.a.s.l. This consisted of high rate (10 Hz) measurements of three-dimensional winds, motion and dry mixing ratio of atmospheric CO_2_ using state-of-the-art spectrometers. Combined with standard underway meteorological ship's navigational parameters this allowed the direct computation of eddy covariance fluxes of momentum, sensible heat and CO_2_ [10,11].

#### (ii) Process studies

O/E incorporated several elements of fieldwork focused on specific Southern Ocean processes. On JR17001 a process study incorporating observations from ship, gliders and aircraft sought to examine the submesoscale and high-frequency processes influencing the evolution of the Southern Antarctic Circumpolar Current Front (SACCF), as well as the surface mixed layer (ML). An array of subsurface autonomous gliders (two BAS and two NOC Teledyne Webb Research Slocum vehicles, and one University of Gothenburg Hydroid Seaglider) was deployed in the southern Drake Passage (figures 1 and 4), alongside a Liquid Robotics SV2 WaveGlider surface vehicle (University of Newcastle). These measured conductivity–temperature–depth (CTD), dissolved oxygen (DO), chlorophyll, backscatter and shear/temperature microstructure. The gliders were configured in either submesoscale (10 km) or mescoscale (100 km) configurations and their paths aligned approximately perpendicular to the sea surface height (SSH) gradient and sampling from the surface to 1000 m depth. Despite difficult weather conditions, a single MASIN aircraft (see below) overflight was made of the region from a height of 25–40 m at a high spatial resolution of 1 km. JR18004's study was focused on the role of topographic mixing over the Discovery Bank of the South Scotia Ridge (figure 1) where a combination of CTDs, vertical microstructure profiler (VMP) profiles, EM-Apex floats and autonomous glider observations were used to investigate in detail the previously observed Taylor Columns and exchange and mixing of waters from the Weddell and Scotia Seas [12].

#### (iii) Moorings

A pre-existing array of moorings in Orkney Passage (fig. 1 [13]) was redeployed as part of O/E. These six moorings provide direct observations of dense water export through the major pathway between the Weddell and Scotia seas. One of these moorings was provided by Lamont–Doherty Earth Observatory with funding from the NOAA Climate Program Office's Ocean Observing and Monitoring Division, who also maintained two additional moorings south of Orkney Plateau. In 2015–2017, additional instruments, and a seventh mooring in Orkney Passage, were also contributed by the NERC-funded Dynamics of the Orkney Passage Outflow (DynOPO) project.

#### (iv) Aerial campaigns

To complement the enhanced ship-borne air–sea exchange observations O/E incorporated approximately 94 h of Meteorological Airborne Science INstrumentation (MASIN) flights. The primary science objective for the aircraft campaign was the characterization of the atmospheric boundary layer and atmospheric surface fluxes over the Southern Ocean open water and sea ice areas. The BAS-operated aircraft incorporated instrumentation to measure the air–sea fluxes of sensible heat, latent heat, momentum and CO_2_ (using a NOC Picarro sensor) using the eddy covariance method, in addition to measurements of mean winds, air temperature, water vapour and incoming and outgoing long and short wave radiation [14,15]. It undertook three field campaigns, operating within the surface boundary layer (20–50 m altitude) and atmospheric boundary layer (50–1500 m) over the northern and southern limbs of the Antarctic Circumpolar Current (ACC), as well as over full and partial ice cover in the Weddell Seas and western Antarctic Peninsula (electronic supplementary material, figures S1 and S2).

### (b) Modelling methodology

#### (i) Regional high-resolution modelling

Two high-resolution regional ocean models were developed. Both are circumpolar configurations of NEMO, the Nucleus for European Modelling of the Ocean, coupled to LIM3, the Louvain-La-Neuve sea ice model [16,17]. Both were run at 1/12° to allow for a vigorous mesoscale eddy field and extend from the Antarctic continent to north of 30°S. One, developed primarily by BAS [18], incorporates a z-star [19] vertical coordinate system, while the other (developed by NOC) uses a hybrid σ-z vertical coordinate scheme (e.g. [20]) and includes ice shelf cavities. These choices were informed by their primary objectives: the z-star model emphasizes Southern Ocean processes north of the subpolar regime, while the σ-z model was envisaged to investigate dense Antarctic Bottom Water (AABW) formation and export, especially within the Weddell Sea. The z-star model incorporated two control runs, one using CORE2 normal year forcing (NYF) [21] and the other run with JRA55-do interannually varying forcing [22]. The hybrid σ-z model was only run with the CORE2 NYF, not JRA55-do because of issues with interactions between the NEMO code and the MPI libraries provided on the ARCHER-2 supercomputer on which the model was run. From these control runs various perturbation experiments, with modified surface winds and buoyancy fluxes, were designed to examine the Southern Ocean response to forcing and provide context to observational analyses. Further details of the model setup and design can be found in [18].

#### (ii) Adjoint modelling

Adjoint modelling using the 1° resolution ECCOv4 [23] was also incorporated into O/E. ECCOv4 is an ocean state estimate, meaning that it has been adjusted to minimize the misfits between the model state and a suite of observations from various sources from 1992 until 2017 (latest release); its adjoint allows the sensitivity of the ocean state to different conditions to be investigated. This tool was used to examine the formation sites and export pathways of SubAntarctic Mode and Antarctic Intermediate Waters (SAMW/AAIW), as well as to innovatively guide perturbation experiments using the z-star model (see Results section).

## 3. Results

At the time of writing, the funded period of O/E has only recently finished (March 2022), and while some elements of work, notably the hydrographic inversions, are ongoing, a significant body of research and several key advances have already emerged in the literature. In this section, we present a brief overview summarizing the key scientific outcomes to date. Several new results are also presented, either updating earlier published works or as novel results in their own right.

### (a) Air–sea heat and carbon fluxes

O/E air–sea flux work had three main foci; filling in the flux ‘data desert’ of the South Atlantic with new observations, developing new analysis products to improve bulk flux formulation, and the examination of flux impacts on ocean properties in models, notably the drivers of existing biases in CMIP5 models.

#### (i) Ship and aircraft observations

Three aircraft campaigns were flown in 2017, 2019 and 2021 for a total of 28 science missions, three of which overflew the JCR, allowing a unique comparison of direct flux measurements between surface and airborne platforms in the Southern Ocean. The ship observations (figure 2) were largely over the open water, and had the advantages of being: (i) long duration (approx. 5000 h of flux measurements) and (ii) close to the surface and thus always measuring the surface flux. By contrast, the aircraft observations were able to measure fluxes in difficult to access regions over sea ice in the Weddell and Bellingshausen Sea as well as over open water, and also to observe the ‘instantaneous’ spatial variations in atmospheric structure. The flights in the vicinity of the JCR enable the comparison of results from different observational platforms. Three low-level flight tracks at 25 m, 26 m and 37 m altitude were flown across the ship track in February 2019 to compare atmospheric pressure, wind speed, friction velocity u*, surface and air temperature and sensible heat flux. Although available data were too sparse for a statistically rigorous comparison, all aircraft and surface system atmospheric parameters show a good correspondence, as does the comparison between eddy covariance and bulk formula derived fluxes (electronic supplementary material, figures S1 and S2). The agreement shown here provides a basis for exploring the integration of aircraft flux measurements over sea ice into wider surface datasets as well as for the interpretation of the aircraft flux measurements as a function of height. This comparison, while not statistically robust, is an important step forward in integrating the disparate surface flux datasets gathered over the Southern Ocean, and beginning to fill in the huge regional data desert in these critical parameters.

This O/E air–sea flux observations and analysis have also provided: the first Southern Ocean evidence for the suppression of CO_2_ gas exchange velocity due to the presence of surface surfactants [24], an important finding particularly for Earth system models; information on the role of ice melt in air–sea CO_2_ flux estimates [11,25,26]; the first global synthesis of air–sea CO_2_ gas exchange velocity measurements derived from eddy covariance, demonstrating that earlier and widely used geochemical tracer-based CO_2_ exchange estimates were likely underestimated at low wind speeds [27]; and the discovery that SST variability in the Southern Ocean demonstrates scale-invariant (fractal) properties, making it plausible that SST can be statistically extrapolated from coarser to finer spatial scales [28].

O/E also developed community analysis tools. Due to the scarcity of direct measurements of the fluxes, different bulk formulae parameterizations of the air–sea turbulent fluxes of sensible heat, latent heat and momentum often display significant disparities. To facilitate the analysis of air–sea fluxes an open-source software package ‘AirSeaFluxCode’ was developed to calculate the fluxes from mean variables (air temperature, sea surface temperature, atmospheric pressure, humidity) [29]. AirSeaFluxCode includes ten different published parameterizations, allowing the results from different parameterizations to be compared directly without the need to implement the original codes. AirSeaFluxCode also outputs height- and stability-adjusted mean meteorological variables. Extensive testing of this package revealed that certain combinations of factors, notably extreme wind speeds, could cause large differences in fluxes which may exceed the limits required for many applications [30,31].

**Figure 3.**
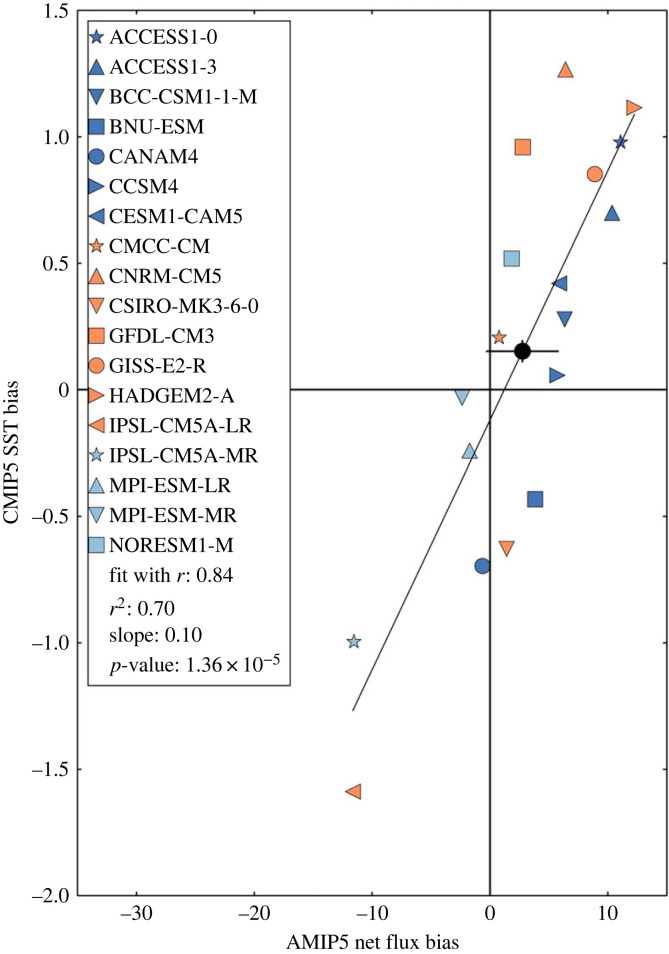
Linear regression of CMIP5 SST historical biases (relative to ERA-interim) onto AMIP5 net flux biases averaged over 40–60°S. The multi-model mean values are plotted with a solid black circle with a cross indicating their estimated observational uncertainties. Reproduced from Hyder *et al*. [33]. (Online version in colour.)

O/E also included significant collaboration with the UK Met Office on the representation of the Southern Ocean in historical and future projections of the CMIP5 and CMIP6 ensembles. A persistent problem in CMIP5, still troublesome in CMIP6, is the presence of a surface warm bias over the Southern Ocean [32]. O/E researchers showed that a significant fraction of this bias can be explained via excessive downward air–sea heat fluxes [33]. This is apparent via the strong (*r* = 0.84) and significant (*p* = 1.36 × 10^−5^) correlation between SST biases in the CMIP5 historical runs and the AMIP5 net surface flux biases (figure 3). The flux bias was shown in other studies to be due to shortwave flux errors, likely associated with poor representation of clouds over the Southern Ocean (e.g. [34]). This work directly informed the development of next generation coupled climate models at the UK Met Office.

**Figure 4.**
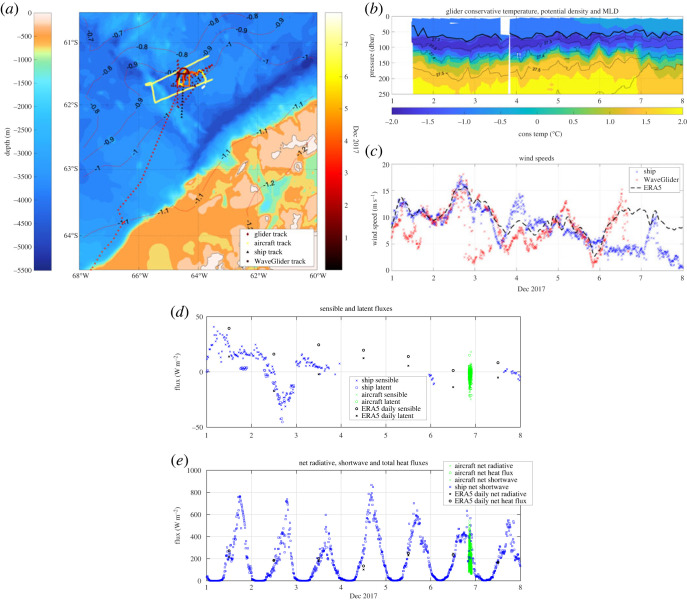
(*a*) Ship track, aircraft track, WaveGlider track and glider track, coloured by day for the first 8 days of the deployment in 2017. GEBCO bathymetry is contoured in metres, alongside mean sea surface height (in dyn.m) at the time of the coordinated glider/ship/aircraft experiment. (*b*) Upper 250 m conservative temperature (°C). (*c*) Comparison of wind speeds observed by ship (U10), WaveGlider (2 m) and ERA5 (U10). (*d*) Sensible and latent heat fluxes (positive into ocean) from the ship measurements, aircraft and daily ERA5 estimates. Strong positive sensible and latent heat fluxes occur around the time of the high wind event on 2 December. (*e*) ML shoaling and increase in ML heat content are driven by the strong positive radiative flux, which dominates the surface heat budget (ERA5 daily, ship and air-derived terms shown). (Online version in colour.)

### (b) Heat and carbon uptake and exchange with the ocean interior

#### (i) Surface mixed layer evolution process studies

O/E sought to link air–sea fluxes to upper ocean evolution through a coordinated field campaign aimed at understanding the submesoscale and high-frequency processes by which surface fluxes influence the development of upper ocean stratification. Previous studies in other regions of the Southern Ocean have highlighted the important role of winds in the generation and sharpening of submesoscale ML fronts (e.g. [35,–37]), which in turn strongly influence the de- and re-stratification of the ML and ventilation of the interior over the seasonal cycle. To further investigate these processes and their role in ML evolution measurements of surface meteorology and turbulent fluxes were acquired simultaneously from the JCR, a constellation of autonomous vehicles and a single MASIN overflight (see Methods).

The initial 8 days of the experiment are displayed in figure 4. During this period, a gradual shoaling of the ML of the ocean was observed from around 70 m on 2 December to 50 m on 7 December, accompanied by a warming of both the ML, and, to a lesser extent, the remnant Winter Water layer below (figure 4*b*). In addition, higher frequency variability in ML depth between individual profiles was also observed, with a typical magnitude of 10–20 m. The meteorological conditions and fluxes accompanying this ML shoaling are shown in panels (*c*–*e*). General agreement is observed between surface wind speeds derived from the ship-based anemometer and the anemometer on the surface vehicle, though some underestimation of the U10 speed is noted, due in part to its shorter mast and in part to spatial separation later in the period (figure 4*a*). The ERA5 reanalysis shows impressive agreement with *in situ* measurements, though it misses some of the largest peak values (e.g. on 2, 5 and 6 December). The strong westerly wind on 2 December coincides with a peak in ocean-to-atmosphere surface and sensible heat fluxes, though these remain modest throughout the observation period compared with the net radiative fluxes (figure 4*e*). Daily average radiative fluxes are between 100 and 300 W m^−2^, driven by the strong shortwave component, and form the dominant term in the net heat flux at the ocean surface. This increases the near-surface stratification and heat content observed by the underwater gliders. While the aircraft measurements provide only a single snapshot of fluxes, they underscore the very high-frequency variability (largely spatial in this instance) in radiative fluxes not captured in reanalysis products such as ERA5, with strong variations dependent on diurnal variability, sea ice coverage and the amount and type of cloud cover. These data will be used to investigate the representation and parameterization of radiative and turbulent fluxes as effective values in numerical models and form the basis for ongoing analysis of meso- and submesoscale ML and frontal evolution.

#### (ii) Subantarctic mode water pathways and variability

O/E also examined larger scale surface to interior ocean processes on the northern side of the ACC. SAMW formation and subduction is one of the main mechanisms by which heat and carbon are sequestered from the atmosphere and exported from the Southern Ocean [6]. Until recently only mean states [38] or linear trends in SAMW formation [39] have been observable. However, with the maturation of the Argo observing array, the O/E project has been a leading contributor to the emergence of a new view of SAMW spatial and temporal variability, and the response of this critical water mass to surface forcing (see also Tamsitt [40]).

Building off earlier SAMW variability analysis [41], Meijers *et al*. [42] used the newly developed O/E optimally interpolated mapping of Argo and ship hydrographic data [43] to show that the two main ‘pools’ of deep SAMW formation in the central and eastern Pacific [38] varied significantly in depth and heat content from year to year. Additionally, they found that these pools typically acted in opposition to one another, forming a ‘see-saw’ dipole such that when the central pool was anomalously deep the eastern pool was anomalously shallow and vice versa. This work also showed that both the Southern Annual Mode (SAM) and El Niño Southern Oscillation (ENSO) were significantly correlated with the dipole pattern with matching phases. It demonstrated that lower frequency (multi annual) variability at SAMW formation sites could be accounted for by the relative phases of these two primary atmospheric modes (electronic supplementary material, figure S3). O/E members also contributed to parallel studies [44,45] using *in situ* observations from the Southern Ocean Flux Station (SOFS) and Ocean Observatories Initiative (OOI) moorings that demonstrated the importance of local turbulent heat flux events driven by southerly wind events in setting ML depths. This idea was further developed by Cerovečki & Meijers [46] who showed that the ML depth dipoles in the SAMW formation regions of both the Indian and Pacific basins are driven by local maxima in mean sea-level pressure (MSLP) variability located between the dipole lobes. These alternately draw anomalously warm (cool) air over their western (eastern) lobes during low (high) phases, leading to locally enhanced surface warming (cooling) and shallow (deep) winter MLs. While the Pacific and Indian dipoles tended to be in phase with one another, this study also showed that strong ENSO years could disrupt this circumpolar coherence.

**Figure 5.**
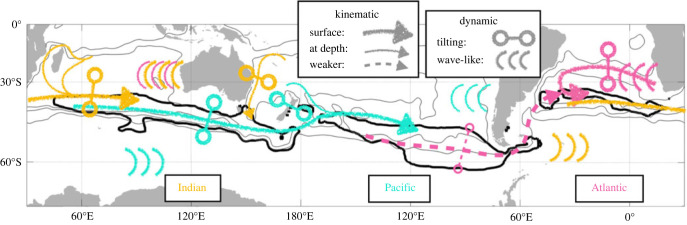
Schematic illustrating the main kinematic and dynamic sensitivities up to approximately 5 years lag for mode water formation regions (MWFRs) mixed layer properties in all three basins: Indian (yellow), Pacific (cyan) and Atlantic (pink). Thick black contours show the median location MWFRs and grey contours the −17, 0 and 30 Sv mean barotropic streamlines. Arrows indicate paths of kinematic sensitivities, with thinner lines indicating paths only found at depth and dashed lines showing relatively weaker paths. The circles connected by lines indicate where dynamic sensitivities resemble dipoles, where a change in isopycnal gradient will affect the MWFRs (the exact location of the symbols is not meaningful). Groups of curves indicate where wave-like patterns are found (from Boland *et al*. [48]). (Online version in colour.)

As the heat and carbon content of SAMW is critical for Southern Ocean export [39], adjoint model analysis was used in O/E to investigate the sensitivity of SAMW formation and export pathways [47] to changes in surface forcing. Boland *et al*. [48] demonstrate that the heat content of these regions is, in all three basins, most sensitive to same-winter, local heat fluxes. While this result aligns with the observational studies above, the adjoint approach also revealed that local and remote wind from one to at least 8 years previously were also important in setting mode water formation site heat uptake. Furthermore, this technique demonstrated that mode water properties responded to gyre advection, wind-driven isopycnal tilt, and even remote coastal waves (figure 5). Consistent with this, Jones *et al*. [49,50] found that the properties of the subducted water in the Southeast Pacific, which ultimately ventilates the subtropical thermocline, are strongly affected by local wind stress over the subtropical gyre.

O/E also supported the development of a new adjoint model's ability to define objective functions in isopycnal space, rather than fixed depth levels [51]. This approach allows model interpretation to more closely follow traditional observational approaches and avoids complexities that can arise when using fixed depth levels to define SAMW (e.g. [49]). Work is ongoing using this technique to quantify the relative importance of remote advection and forcing versus local winter heat loss in the Pacific and assess the sensitivity of the volume of mode water formation regions to forcing and advection.

### (c) Southern Ocean interior change and export

#### (i) South Atlantic and Weddell Sea properties and change

**Figure 6.**
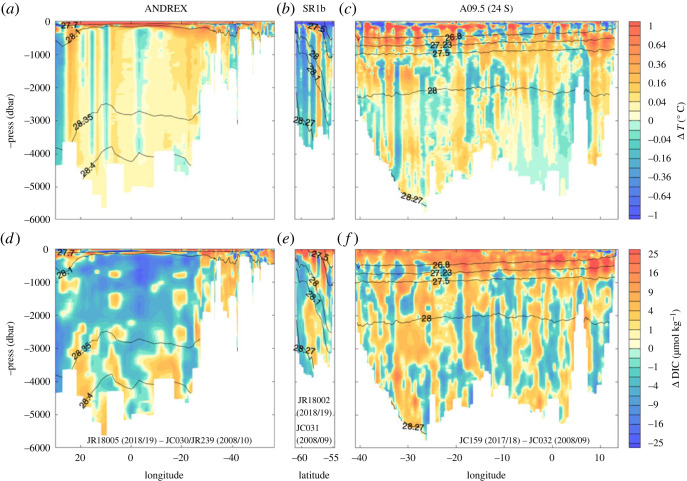
Approximately decadal (2017/2018–2008/2010) difference between O/E hydrographic ‘box’ boundary sections (figure 1) of potential temperature (°C, (*a–c*)) and DIC (µmol kg^−1^, (*d–f*)) for 24°S (*a*,*d*), SR1b (*b*,*e*) and ANDREX (*c*,*f*) sections. Voyages and dates as labelled (see electronic supplementary material, table S1). Previous decade observations detailed in [7,9]. (Online version in colour.)

O/E has produced a milestone set of observations of the full-depth heat and carbon properties and export over the South Atlantic and Weddell Sea. Figure 6 shows the change in potential temperature and dissolved inorganic carbon (DIC) on the sections collected by O/E surrounding the South Atlantic and Weddell boxes (figure 1) relative to occupations approximately a decade earlier. They reveal clear surface-intensified warming over the full depth of the northern boundary of the Weddell Sea (ANDREX section), where DIC has decreased in the Circumpolar Deep Water (CDW) of the eastern Weddell Sea but increased in the more recently exposed AABW and surface layers. This warming of most of the water column is consistent with water mass heave induced by a reduction in underlying AABW volume (see below). The sign change of DIC is also consistent with this, but until the further budgetary analysis is complete we cannot be certain that flux changes have not also influenced the DIC on the ANDREX line. Across the ACC, the previously observed tendencies for cooling south of the Polar Front and at the surface [52,53], and increasing DIC along with decreasing temperature in AAIW [54] are present on the SR1b section. Comparisons in dynamic height space (not shown) reveal, however, that the decrease in temperature and DIC in the southern ACC here is associated with changes in the ACC front locations rather than changes in properties of the interfrontal zones. At 24°S, surface-intensified warming and increase in DIC are visible behind the eddy variability. Changes in deep and abyssal waters map with the differing connectivity of the basins: the North Atlantic Deep water in the Brazil Basin shows a negligible average temperature trend while the bottom waters are both warming and increasing in DIC (consistent with the longer-term trends [55]), the deep Angola Basin, isolated from the south by the Mid-Atlantic and Walvis Ridges, shows a tendency toward cooling and a smaller average DIC trend. Analysis of these data is ongoing, and will ultimately be used to establish watermass transformation and carbon, heat and freshwater transports, net surface fluxes, storage within the Weddell gyre (following [7,8,56]) and South Atlantic [9,57], and to establish decadal changes in these properties.

**Figure 7.**
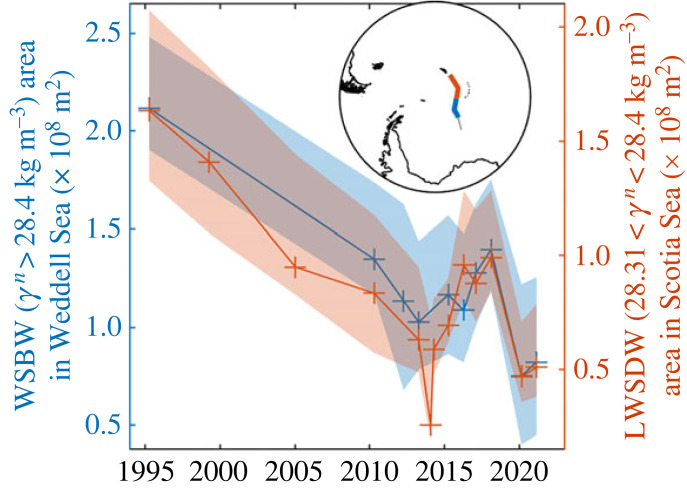
Reduction in WSBW, blue (LWSDW, red) areas on the A23 hydrographic section (see insert) south (north) of the South Scotia Ridge between 1995 and 2021. Shaded areas indicate the uncertainty from instrumentation and varying sampling locations between cruises. Figure adapted and extended from Abrahamsen *et al*. [13]. (Online version in colour.)

**Figure 8.**
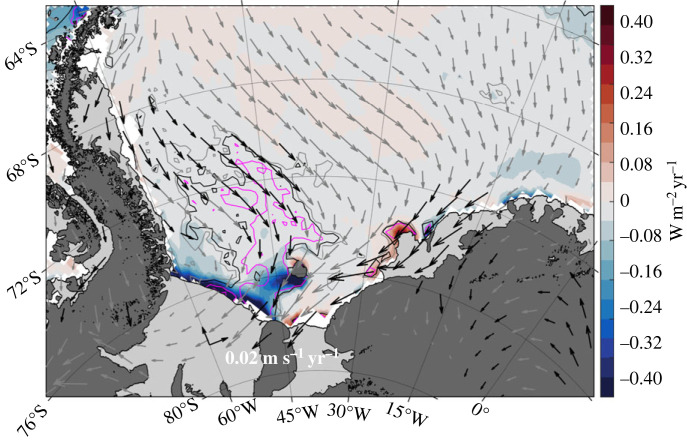
Linear trend in sea ice formation rate showing reduction in front of the Ronne Ice Shelf from 1992 to 2020, estimated for austral autumn/winter (April to October), driven by northerly wind trends over the same period (vectors from ERA5). Statistical significance of over 90%, 95% and 99% in sea ice formation rate trend is highlighted with grey, black and magenta contours, respectively. Vectors with over 90% confidence in either zonal or meridional component trend are in black. Reproduced from Zhou *et al*. [63] (Online version in colour.)

The warming trend observed on the northern boundary of the Weddell Sea (figure 6) agrees with the broader full-depth warming trend within the gyre itself [58] that exceeds the global and regional mean estimates from other O/E studies by a factor of five (e.g. [59,–61]). Early O/E analyses [62] showed that higher frequency Weddell Sea property variability can be attributed to regional changes in winds, but more recent analysis [63] has demonstrated that the long-term warming signal is almost entirely driven by a 30-year reduction of Weddell Sea Bottom Water (*γ_n_* > 28.40 kg m^−3^) volume and the subsequent deepening of overlying isopycnals [64]. O/E [13] showed that overlying water masses have not significantly changed the volume, but the deepening of isopycnals has impacted the volume of Weddell Sea Deep Water (WSDW) able to escape to the Scotia Sea via the Orkney Passage (figure 1). This research showed that the enhanced sampling frequency enabled by O/E could resolve interannual, as well as seasonal [65], variability and noted a partial recovery in dense water areas between 2014 and 2018. Here, we extend this to 2021 and show that this recovery was temporary and Lower Weddell Sea Deep Water (LWSDW) continues to shrink in line with the long-term trend (figure 7). Weddell Sea Bottom Water (WSBW) area has fallen in all available Weddell Sea sections by approximately 30% since 1992. Using the complementary bulk formula and sea ice convergence approaches, O/E, in collaboration with the Horizon 2020 project SO-CHIC, found that the reduction in dense water is accompanied by an approximately 40% decline in sea ice formation rates over the continental shelf in front of the Ronne Ice Shelf. This reduction in sea ice production consequently reduces ocean heat loss, brine rejection and subsequent dense shelf water (DSW) formation [63]. This is the key precursor to WSBW (e.g. [66]) and the observed change in DSW production is sufficient to drive the observed reduction in WSBW volume. The sea ice formation rate reduction is driven by increased northerly winds since 1992 (figure 8) that act to weaken sea ice export from the region. Here the prevailing meridional component of winds is southerly. These winds act to ‘blow out’ newly formed sea ice and maintain polynyas in front of the Ronne Ice Shelf, permitting sustained ocean heat loss and sea ice and dense water production. The increased ‘northerly’ trend is really a weakening of the prevailing southerlies. This retains more sea ice locally and thus opens water areas and ocean heat loss is reduced; consequently, less ice is produced over a winter season. These wind trends may be linked to a general deepening of the Amundsen Sea Low (ASL) during this period. This ASL deepening itself may be due to a negative polarity in the Interdecadal Pacific Oscillation (IPO) since 1992, providing exciting evidence for a chain of causality of teleconnections between natural large-scale climate variability in the tropical Pacific and WSBW volumes and Weddell Sea dynamics.

#### (ii) Tracing freshwater inputs with oxygen isotopes

To fully address O/E's objective of understanding SO heat and carbon storage, export and processes, it is also important to understand freshwater changes, since these can impact on air–sea fluxes of heat and carbon, upper ocean stratification, mixing, and so on. To trace and quantify freshwater inputs of different origin, O/E made extensive use of the oxygen isotope composition (δ^18^O) of seawater, taking advantage of state-of-the-art analytical facilities at the National Environmental Isotope Facility at BGS Keyworth. When measured alongside salinity, δ^18^O enables quantitative partitioning of freshwater inputs and changes into those due to sea ice and those due to meteoric water (glacial melt and precipitation).

Using this approach, Meredith *et al*. [67] produced new insight into the relative importance of different freshwater inputs to the West Antarctic Peninsula region, an area of very strong climatic sensitivity, and how this importance varies across a range of spatial and temporal scales. Collaborating with the French WAPITI team, Akhoudas *et al*. [68] used δ^18^O to reconcile different quantifications of AABW production and export produced previously using contrasting techniques. Meredith *et al*. [69] used repeat δ^18^O data from the A23 section to examine year-on-year changes in sea ice and meteoric water input. They found a particularly strong pulse of sea ice melt injected to the surface of the northern Weddell Sea, following the unprecedented decrease in sea ice extent in 2016. They also traced the strong injection of meteoric water from the melt of the A68 megaberg at the northern end of the Scotia Sea adjacent to South Georgia, and determined the complex spatial structure of the meltwater released.

#### (iii) Ocean heat content sensitivity to wind stress

Several experiments were run using the O/E high-resolution z-star model (see Methods), described in Munday *et al*. [18], to investigate how changing boundary conditions can impact Southern Ocean heat storage, export and processes. That study also demonstrates that the neglect of the difference between wind velocity and ocean velocity in bulk formula flux calculations can lead to an approximately 50% increase in surface eddy kinetic energy (EKE) over the Southern Ocean in high-resolution models. This is despite a modest approximately 2% change in the mean zonal wind stress and approximately 10% change in the power input from the wind to the ocean. The extra EKE acts to flux heat poleward and influences circumpolar sea-ice cover in all seasons. The importance of eddies in Southern Ocean heat fluxes reinforces the findings of an O/E assessment underscoring the importance of regional eddy variability in modelled responses to winds [70]. Below we describe two additional results emerging from ongoing analysis of Southern Ocean heat uptake using this model.

**Figure 9.**
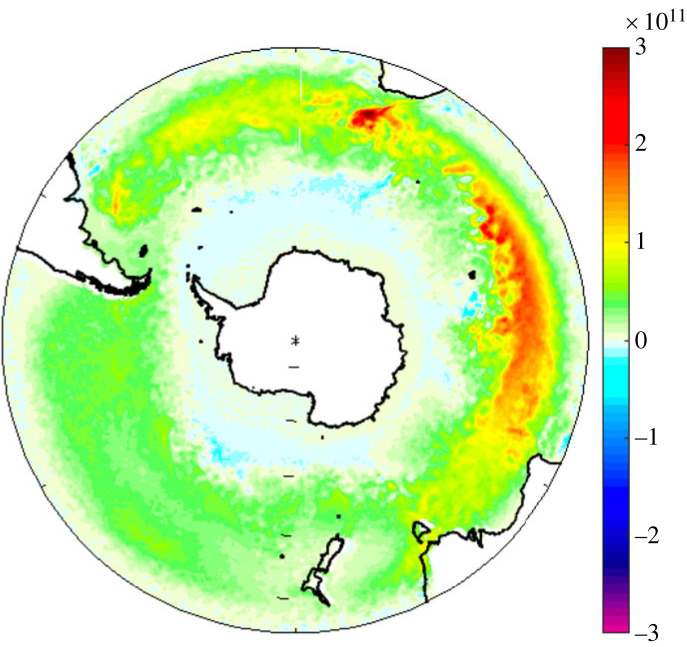
Difference (JRA55TAU–JRA55IAF) in September mean equatorward Ekman heat transport between the perturbed wind stress and control model experiments showing asymmetric heat transport across the Southern Ocean basins. Units W per model grid cell. (Online version in colour.)

The sensitivity of Southern Ocean heat transport to increasing Southern Ocean wind stress was investigated using the control run forced by JRA55-do interannually varying forcing for 40 years (referred to as JRA55IAF) and a run with perturbed wind stress increased by 10% in the latitude range 60–40°S and tapered to control run values over 10° on either side (referred to as JRA55TAU). Investigation of ocean heat transport in the two runs shows longitude-dependent differences in the response of the heat transport to increasing wind stress. Specifically, the equatorward Ekman heat transport increase over a narrow band at about 45–40°S is notably stronger in the Indian Ocean sector of the Southern Ocean compared to the Atlantic sector which in turn is stronger than the Pacific sector (figure 9). The integrated Ekman heat transport across 40°S in the Indian Ocean sector is 0.72 PW in JRA55TAU compared to 0.58 PW in JRA55IAF, an increase of 0.14 PW. The increase reflects the combined effects of changes in both the wind stress and the temperature difference between the Ekman layer and the water column as a whole.

Ongoing work is investigating the relative contributions of the wind stress and temperature difference terms, together with the implications for regional temperature changes. In particular, the narrow meridional extent of the pattern implies a strong heat transport convergence in the Indian Ocean sector (and to a lesser extent the Atlantic sector) at 40°S. This suggests that strengthening Southern Ocean winds could lead to longitude-dependent differences in the upper ocean. The results presented here indicate that intensification of the winds may have played some role in recently reported buoyancy-driven increases in zonal flow north of the Subantarctic Front [71], and further analysis is planned to determine the size of this contribution.

**Figure 10.**
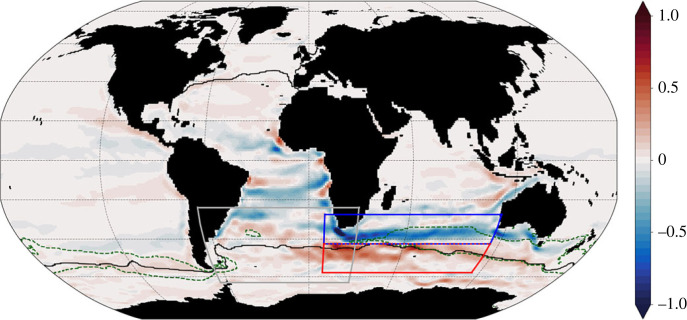
Adjoint model sensitivity to zonal wind stress of the mean temperature of the O/E box (grey box) in °C/(N m^−2^). The time-average 0.25 m SSH contour (black line) and 300 m mixed layer depth contour (dashed green line) in August highlight the relative positions of the northern ACC edge and mode water pools. The blue and red lines are where zonal wind (stress) anomalies are applied to produce a change in the mean temperature of the O/E box. (Online version in colour.)

The second ongoing experiment uses a semi-optimal perturbation approach to examine the response of South Atlantic heat content to zonal wind stress changes. This approach uses the significantly computationally cheaper ECCOv4r3 adjoint experiment to first produce a time series of adjoint sensitivities, which permit a quantitative assessment of where/how to perturb the zonal wind stress in a way that will produce the largest impact on a chosen metric. In this instance, the adjoint calculation evaluates the mean temperature of the South Atlantic O/E box (figure 1) and describes how changes to the zonal wind stress are expected to influence this mean temperature. Figure 10 highlights the position of the O/E box relative to the sensitivity pattern at a lag of around 8 years and how this aligns with the circulation of the forwards ECCOv4r3 model.

The bands of strong positive/negative sensitivity to the east of the O/E box are persistent at most lags. These bands are fairly local to the region itself, or spread to the east, north of or immediately over the ACC.

The red and blue boxes in figure 10 are the regions chosen to receive wind stress perturbations. In the blue (red) box, a decrease (increase) in wind stress will lead to an increase in the O/E box's mean temperature. Two sets of perturbations were designed; the first has the perturbations as just described while the second reverses the wind stress changes to induce a decrease in the O/E box's mean temperature. In addition, the applied zonal wind stress perturbation was also convolved with the adjoint sensitivity fields. This produces the change in the O/E box mean temperature that the adjoint model would predict for the specific perturbation experiment. [48]

**Figure 11.**
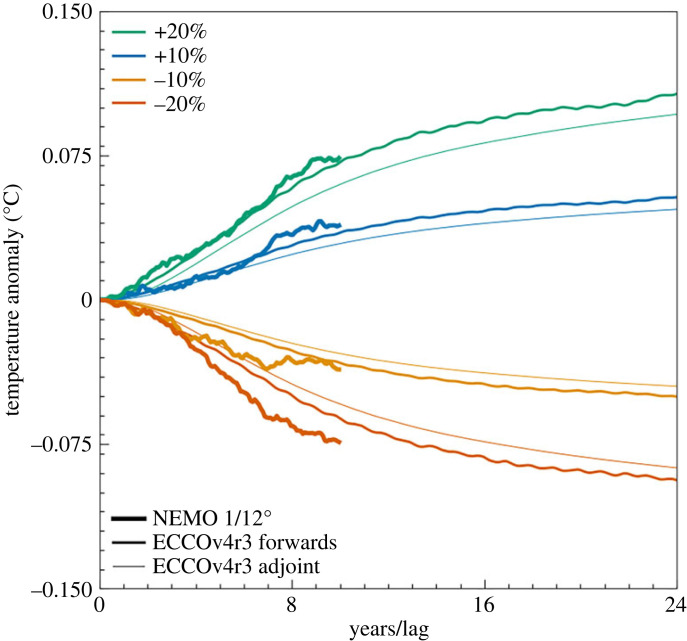
Time series of temperature as an anomaly from the relevant control. Heavy lines: NEMO 1/12° as an anomaly from JRA55IAF. Medium lines: ECCOv4r3 forward runs as an anomaly from the standard ECCOv4r3 forward run. Light lines: convolution of ECCOv4r3 adjoint sensitivities with the wind stress anomalies used for the forward perturbation experiments as a function of lag. Lag is measured from the end of the adjoint run. This time series has been inverted to provide a better visual comparison. (Online version in colour.)

The mean temperature of the O/E box for both NEMO 1/12° and the ECCOv4r3 forwards runs are compared with the adjoint convolution in figure 11 as an anomaly from the relevant control run. All model time series are remarkably similar and follow the change in mean temperature predicted by the adjoint sensitivities. All four NEMO 1/12° perturbation experiments match the magnitude of both the ECCOv4r3 forwards run and adjoint sensitivity convolution well, with only relatively small differences due to the nonlinear effects present in the forward runs, or the difference in their forcings. This work demonstrates the utility of this semi-optimal approach to defining perturbation experiments and is being developed further to understand the heat uptake and storage efficiency of the South Atlantic region.

#### (iv) Model representation of subpolar water mass properties

**Figure 12.**
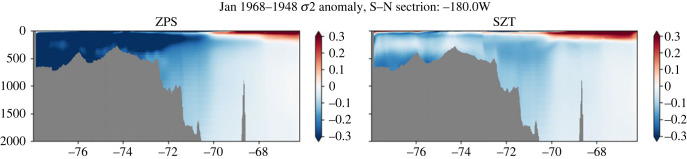
Density (σ_2_, kgm^−3^) biases after 20 years of model integration along a meridional section at 180°W, through the Ross Sea. Left (right) panel shows biases in the ZPS (σ-z) simulation. (Online version in colour.)

Accurate representation of water mass properties on the Antarctic shelves is challenging, but these water mass properties have significant consequences for the formation of AABW and ocean ventilation (e.g. [72]). Conventional z-level numerical models suffer from spurious diapycnal mixing [73], adversely affecting their ability to accurately represent the way in which dense water masses overflow from shallow seas into the deep or abyssal ocean [74]. Terrain-following (σ) coordinates are able to better represent the natural flow of these dense waters, though they can be subject to potentially large pressure gradient errors [20]. O/E explored the potential to improve high-resolution model representation using a hybrid σ-z vertical coordinate, in which the vertical coordinate transitions from z- to σ- where the ocean depth becomes shallower than 1500 m. Two 20-year simulations of the Southern Ocean configuration of NEMO forced with the CORE2 normal year dataset, one using z partial steps (ZPS) and one using σ-z are compared. Both configurations use the same atmospheric forcing dataset and bulk formulae. Any differences in heat and freshwater fluxes into the ocean are associated with different ice cover and sea surface temperatures resulting from the different coordinate representation. The comparison shows consistent reduction in the density drift on the Antarctic shelves when using the hybrid coordinate (figure 12). The most pronounced improvements are in the Ross Sea, and to a lesser extent the Weddell Sea. Density (σ_2_) biases along a section at 180°W exceed −0.4 kg m^−3^ on the Ross Shelf in the ZPS simulation, while in the σ-z simulation, the biases are generally below −0.1 kg m^−3^. Temperature biases evolve similarly in the two configurations, with both models cooling by 1–1.5°C. The difference in density biases is primarily a result of a strong freshening of 0.5–1 PSU on the Ross Shelf in the ZPS simulation. The σ-z simulation also freshens but only by 0.1–0.4 PSU. These lessened biases over the Antarctic continental slope improve the AABW volumes of the simulations. Over a box enclosing the Weddell Sea and the Atlantic, AABW volumes remain steady over the 20 years in the σ-z simulation, while they decline in the ZPS run, falling away by 2–3% (electronic supplementary material, figure S4). The lack of a reduction in dense water volume in the σ-z model is likely due to the improved representation of dense water cascading permitted by terrain-following coordinates and more effective transport of shelf waters to depth. This work will inform ongoing hybrid coordinate system model development at NOC including the Shelf-enabled NEMO (SE-NEMO) configuration being used in the CANARI project, and contribute to future configuration development within the UK Joint Marine Modelling Programme.

## 4. Discussion and Conclusion

### (a) Key scientific outcomes

O/E has made significant advances towards filling in the vast region of poor data coverage around the South Atlantic, Weddell Sea and Antarctic Peninsula, and in particular, has performed a milestone decadal reoccupation of key sections constraining the South Atlantic and Weddell Sea. It has been at the forefront of innovative observations, deploying autonomous eddy covariance heat and carbon flux observations over multiple voyages that has increased the number of direct CO_2_ flux measurements over the Southern Ocean by roughly a factor of five, resulting in improved understanding in air–sea CO_2_ exchange not only at these latitudes but globally. O/E also brought together ship, aircraft and surface and subsurface autonomous vehicles for the first time in the Southern Ocean to make coordinated observations of the high-frequency variability and evolution of air–sea fluxes and their impact on ML evolution. It has continued and enhanced existing observational time series, through higher frequency transect occupations, denser mooring arrays and the expansion of variables observed on these sections, notably including oxygen isotopes. O/E's analyses have delivered important community advances in our understanding of climate model SST biases, SAMW formation and export variability, AABW export trends and the role of ice melt in setting Southern Ocean properties. The models developed have provided new insights into ocean processes, such as mode water variability, eddy dynamics and ocean heat uptake in response to wind stress, and also into vertical coordinate system development and the advantages of combined adjoint-forward model experimental design. This is of course an immensely compressed summary of the activities of several dozens of researchers over 6 years and, for example, O/E also delivered novel work using machine learning to classify Southern Ocean profile types [75], defined a new probabilistic metric for fronts [76], undertook diffusive flux analysis [77], produced chapters in textbooks on subduction [78], satellite altimetry [79] and mixing [80], and even investigated the hydrodynamics of induced CTD package oscillations [81].

Despite being predominantly focused on the physical aspects of the ocean/climate system, O/E research supported numerous biogeochemical or ecosystems studies. Notable among these were important advances in reframing the carbon cycle of the Weddell Gyre as not just a process of vertical overturning, but the interaction between regions of high biological carbon drawdown and the lateral gyre circulation [82], high-profile studies demonstrating the impact of projected Southern Ocean warming on benthic communities [83], and the pitfalls of applying present day ecosystems proxy relationships to future ocean dynamics [84].

### (b) Wider impacts

In addition to the direct advances described above, O/E has provided an underpinning element of fundamental circulation and physics research, as well as platforms, for numerous other scientific collaborations. Most notably it has supported the NERC programme: the Role of the Southern Ocean in the Earth System (RoSES) and its component projects; focused on the biogeochemical elements of the Southern Ocean, carbon processes and their global climate impacts (e.g. Williams *et al*. [85]). It also made significant contributions to parallel UK programmes working in the North Atlantic (e.g. adjoint modelling developed in O/E [86]), coordinated mooring deployments with the DynOPO project and provided three voyages supporting the Transient tracer-based Investigation of Circulation and Thermal Ocean Change (TICTOC) programme measuring CFCs. It has impacted research beyond the UK as a contributor to and supporter of the US SOCCOM and more recently BIOARGO programmes, both through the deployment of floats and scientific collaboration and exchange. Similarly, it has ongoing collaborative links with the Horizon 2020 SO-CHIC (see Sallee *et al*. [87]) and ESA-EU SO-ICE projects and has produced important advances in our understanding of Weddell Sea bottom water evolutions. These projects have provided a collective focus on Southern Ocean heat and carbon uptake, and have stimulated community engagement and momentum through numerous conference sessions, discussion groups and ultimately the present Royal Society Discussion meeting and proceedings, collectively convened by O/E, RoSES and SO-CHIC. O/E has also supported dozens of PhD students from across the globe through both direct involvement with O/E as well as collaboratively via the above projects and the provision of berths on research voyages.

Finally, O/E's impact has been felt by numerous policy facing and scientific coordination bodies. Its research featured heavily in the IPCC SROCC Chapter 3 Polar Oceans, led and contributed to by O/E researchers [88]. Similarly, the IPCC AR6 Chapter 9 [89] incorporated advances delivered by O/E over the prior 5 years. Other assessments, such as the BAMS state of the climate report have been led and co-authored by O/E researchers, incorporating O/E observations and products since 2016. O/E researchers provided key polar support to UK Government policy and led the delivery of the UK State of the Polar Oceans assessment report and supporting events such as the United Nations' CoP26.

O/E research and researchers have significantly contributed to and helped guide the Southern Ocean Observing System (SOOS), notably in the development of its science implementation plan [90] and contributing to key working groups and community white papers [31,91]. It has supported the GO-SHIP programme by delivering cruises, observations and expertise [92,93] and its expertise has been directly incorporated into ecosystem service assessments [94], fisheries management [95] and supported bodies such as the Commission for the Conservation of Antarctic Marine Living Resources (CCAMLR)'s Integrating Climate and Ecosystem Dynamics in the Southern Ocean (ICED) programme.

### (c) Future work

While O/E's funding has finished, there are numerous ongoing threads of analysis and development. Prominent among these is the analysis of the South Atlantic and Weddell Sea budgets, as defined by the O/E hydrographic sections. This will provide a milestone assessment of the heat and carbon budgets within this region, as described in the results section. Particularly useful in this analysis will be the O/E δ^18^O data, which will help elucidate the thermohaline transformations within the Weddell Gyre driven by recent variations in sea ice and glacial melt. Following earlier approaches [96] these will attribute circumpolar-scale salinity stratification and zonation to different combinations of precipitation, glacial melt and sea ice melt. δ^18^O sample collection is continuing and being enhanced, especially via the recently commenced ‘Biogeochemical processes and ecosystem function in changing polar systems and their global impacts’ (BIOPOLE) programme, which will in particular enable new information on the biogeochemical and biological impacts of different freshwater inputs to be ascertained. BIOPOLE will also carry on other elements of O/E, notably maintaining the A23 hydrographic section, Orkney Passage moorings and extending the autonomous subsurface glider-based analysis of ML evolution into marginal and under sea ice zones. The value of such high-resolution autonomous measurements for improving parameterizations of ML development has already been demonstrated in non-polar oceanographic contexts (e.g. at the North Atlantic OSMOSIS array), with observations feeding directly into validating large eddy simulations (e.g. [97]) and ultimately improving the representation of submesoscales in climate models (e.g. [98] and references therein). BIOPOLE will also extend the ongoing O/E CMIP6 water mass analysis to incorporate nutrient export from the Southern Ocean.

Analysis of the AABW trends and export pathways identified by O/E will continue under SO-CHIC, but also expand to draw in the influence of the Ronne-Filchner Ice Sheet via SO-ICE as well as investigate previously unmapped pathways of AABW export identified via the ANDREXII section at the South Sandwich Trench under the upcoming ‘Ocean–Cryosphere Exchanges in ANtarctica: Impacts on Climate and the Earth System’ (OCEAN:ICE) Horizon Europe project.

O/E has demonstrated the power of coordinated large-scale cross-centre research projects to deliver significant advances across multiple disciplines, generating a holistic analysis and understanding of the Southern Ocean that would be impossible for any single centre to deliver alone. The datasets, models and advances in understanding delivered by O/E will continue to shape Southern Ocean research for decades to come.

## Data Availability

This is a summary article for a large project including dozens of individual studies and almost 100 published papers. Where articles are cited in reference to data and results, please see the referenced article and their data accessibility statements. All O/E datasets as well as cruise reports are (or are being made) freely available via the British Oceanographic Data Centre (https://www.bodc.ac.uk/data/bodc_database/nodb/data_collection/6618/) with a dedicated data manager (co-author R.P.O.). O/E model and MASIN flight data are similarly archived at the Centre for Environmental Data Analysis (https://catalogue.ceda.ac.uk/uuid/c21cf5fb28c84cb2820a4785c503ea8c). The data are provided in electronic supplementary material [99].
